# Formation of Core-Shell Nanoparticles Composed of Magnetite and Samarium Oxide in *Magnetospirillum magneticum* Strain RSS-1

**DOI:** 10.1371/journal.pone.0170932

**Published:** 2017-01-26

**Authors:** Hirokazu Shimoshige, Yoshikata Nakajima, Hideki Kobayashi, Keiichi Yanagisawa, Yutaka Nagaoka, Shigeru Shimamura, Toru Mizuki, Akira Inoue, Toru Maekawa

**Affiliations:** 1 Bio-Nano Electronics Research Centre, Toyo University, Kawagoe, Saitama, Japan; 2 Graduate School of Interdisciplinary New Science, Toyo University, Kawagoe, Saitama, Japan; 3 Japan Agency for Marine-Earth Science and Technology, Yokosuka, Kanagawa, Japan; Brandeis University, UNITED STATES

## Abstract

Magnetotactic bacteria (MTB) synthesize magnetosomes composed of membrane-enveloped magnetite (Fe_3_O_4_) or greigite (Fe_3_S_4_) particles in the cells. Recently, several studies have shown some possibilities of controlling the biomineralization process and altering the magnetic properties of magnetosomes by adding some transition metals to the culture media under various environmental conditions. Here, we successfully grow *Magnetospirillum magneticum* strain RSS-1, which are isolated from a freshwater environment, and find that synthesis of magnetosomes are encouraged in RSS-1 in the presence of samarium and that each core magnetic crystal composed of magnetite is covered with a thin layer of samarium oxide (Sm_2_O_3_). The present results show some possibilities of magnetic recovery of transition metals and synthesis of some novel structures composed of magnetic particles and transition metals utilizing MTB.

## Introduction

Magnetotactic bacteria (MTB) form intracellular chains of magnetosomes, which contain membrane-enveloped magnetic crystals comprised of magnetite (Fe_3_O_4_) or greigite (Fe_3_S_4_) [1,2]. The size of magnetosomes ranges from 35 to 120 nm, and the shape varies depending on the bacterial strains. The size and shape are, however, highly uniform in each strain [3–5]. The biomineralization process is strictly controlled by the magnetosome-related genes. Such a high degree of control of the synthetic process of magnetosomes occurring in MTB has a significant advantage over the other synthetic methods of magnetic nanoparticles particularly in terms of the morphological definition and biocompatibility [6–9]. Magnetosomes are of great importance considering their application to nanotechnology-based biomedical studies; e.g., they can be used as nano magnetic resonance imaging enhancing agents, nano hyperthermic cancer treatment media and nano drug delivery vehicles [10], the performances of which are however totally dependent on the magnetic properties of the magnetosomes.

Uptake of iron occurring in MTB has been extensively studied and it has been shown that the cells have a high affinity and specificity with iron. It has been demonstrated that magnetosomes doped with manganese, titanium, and copper can be synthesized by several MTB thanks to their relatively high affinity with those transition metals, but the mechanism of uptake of transition metals and reproducibility of magnetosomes have not yet been quantitatively understood [11–14]. It is known up to the present that at least three strains; that is, *Magnetospirillum gryphiswaldense* MSR-1, *M*. *magnetotacticum* MS-1 and *M*. *magneticum* AMB-1, form magnetosomes doped with cobalt, which results in an increase in the coercivity of the magnetite nanoparticles [15]. It is supposed that transition metal ions doped in magnetosomes are in the +2 oxidation state and occupy some Fe^2+^ octahedral sites in the Fe_3_O_4_ inverse spinel structure. It was in fact recently clarified that Fe^2+^ ions were replaced by Co^2+^ at the octahedral sites of magnetosome magnetite [16]. It was also demonstrated that *Magnetospirillum* species incorporate copper and manganese into magnetosomes, as a result of which the magnetic properties of magnetite particles alter [17,18].

Herein, we investigate the effect of samarium; a transition metal belonging to lanthanide, on the formation of magnetosomes and any change in the magnetic properties of magnetosomes, knowing that lanthanide ions possess unique optical and magnetic properties associated with 4*f*-electronic configurations. Lanthanide-doped magnetite nanoparticles have been artificially synthesized by several methods such as the coprecipitation, reverse micelle, and thermal decomposition methods [19,20]. There are several reports on the effect of lanthanide metals on the behavior of some bacteria [21–24]. but their effect on MTB has not yet been investigated. Samarium is commercially of great importance [25,26]. All of the lanthanide ions are known to be stable in the +3 oxidation state, whereas samarium, europium and ytterbium can also be in the +2 oxidation state [27]. We therefore suppose that there is a possibility that MTB may incorporate samarium into magnetosomes. In this study, we synthesize magnetite covered with samarium oxide in *Magnetospirillum magneticum* strain RSS-1, which is isolated from sediment in a freshwater environment. The magnetic crystals show some unique core-shell structure composed of Fe_3_O_4_ and Sm_2_O_3_.

## Materials and Methods

### Isolation and growth of strain RSS-1

Sediment together with freshwater, the ratio of which was 1:2, was collected during the autumn season from an agricultural waterway in Kawagoe, Saitama, Japan (35°54’16”N, 139°24’25”E) and transferred to two 1 L plastic bottles. No specific permissions were required for the collection of the sediment and freshwater, where neither endangered nor protected species are involved. MTB were enriched by neodymium-boron magnets (ϕ10 × 10 mm) of 0.48 T, attaching them to the surface of the bottles at 1 cm above the sediment-water interface for 90 min, and then cells accumulated by the magnets were collected with a Pasteur pipette and transferred to a test tube. The cells were magnetically concentrated by a modified MTB trap device for 90 min and after having been inoculated in 15 mL screw-capped glass culture tubes [28], which were filled up with semi-solid agar containing 250 μM ferric (Fe) quinate and 250 μM samarium (Sm) quinate, they were incubated at 28°C in dim light. Bacteria steadily grew for 7 days. Axenic cultures of spirilla cells were then obtained by the modified MTB trap device, followed by dilution for extinction.

The present medium, which was modified from the one used for *Magnetospirillum*; i.e., DSMZ380, was composed of 10 mM HEPES buffer, 0.011 g L^-1^ KH_2_PO_4_, 0.12 g L^-1^ NaNO_3_, 0.37 g L^-1^ tartaric acid, 0.37 g L^-1^ Succinic acid, 0.05 g L^-1^ sodium acetate, 0.5 mg L^-1^ resazurin, 0.05 g L^-1^ cysteine hydrochloride, 0.5 mL L^-1^ mineral solution, and 1.0 g L^-1^ agar mixed with 1 L distilled water. The mineral solution was composed of 15 g L^-1^ nitrilotriacetic acid, 30 g L^-1^ MgSO_4_•7H_2_O, 5.0 g L^-1^ MnSO_4_•2H_2_O, 10 g L^-1^ NaCl, 1.0 g L^-1^ FeSO_4_•7H_2_O, 1.8 g L^-1^ CoSO_4_•7H_2_O, 3.0 g L^-1^ CaCl_2_•2H_2_O, 1.8 g L^-1^ ZnSO_4_•7H_2_O, 0.2 g L^-1^ KAl (SO_4_)_2_•12H_2_O, 0.1 g L^-1^ H_3_BO_3_, 0.1 g L^-1^ Na_2_MoO_4_•2H_2_O, 0.25 g L^-1^ NiCl_2_•6H_2_O, and 3.0 mg L^-1^ Na_2_SeO_3_•5H_2_O dissolved in 1 L distilled water. After pH having been adjusted to 7.0, the semi-solid agar was autoclaved at 121°C for 15 min, and then 1 mL sterile vitamin solution, 2.5 mL sterile Fe quinate and 2.5 mL sterile Sm quinate were added to the medium. The vitamin solution was composed of 20 mg L^-1^ biotin, 20 mg L^-1^ folic acid, 0.1 g L^-1^ pyridoxine hydrochloride, 50 mg L^-1^ riboflavin, 50 mg L^-1^ thiamine, 50 mg L^-1^ nicotinic acid, 50 mg L^-1^ pantothenic acid, 1 mg L^-1^ vitamin B12, 50 mg L^-1^ *p*-aminobenzoic acid and 50 mg L^-1^ thioctic acid mixed with 1 L distilled water. 100 mM Fe quinate and 100 mM Sm quinate were prepared by mixing 45 g L^-1^ FeCl_2_•6H_2_O and 19 g L^-1^ quinic acid, and 52 g L^-1^ SmCl_3_•6H_2_O and 19 g L^-1^ quinic acid with 1 L distilled water, respectively.

The growth of strain RSS-1 in 45 mL liquid medium containing Fe quinate and/or Sm quinate under microaerobic conditions (1% O_2_ + 99% N_2_) was measured using a bacterial counting chamber (Erma).

### Sequence and phylogenetic analysis of strain RSS-1

DNA was extracted from strain RSS-1 using DNeasy (QIAGEN) and the 16S rRNA gene was amplified using the universal bacterial primers 27F (5’-AGAGTTTGATCCTGGCTCAG-3’) and 1492R (5’-GGTTACCTTGTTACGACTT-3’)[29]. The PCR reaction was carried out as follows: the template DNA was initially denatured at 95°C for 2 min, followed by 25 cycles of the temperature control; i.e., 95°C for 20 s, 50°C for 30 s and 72°C for 90 s, and a final extension step at 72°C for 5 min. The PCR product was purified using a QIAquick PCR Purification Kit (QIAGEN) and cloned into the pCR2.1 T vector using a TA Cloning Kit (Invitrogen) and chemically competent DH5α cells (TaKaRa). The transformed cells were incubated overnight at 37°C on LB agar plates with ampicillin. 7 clones were randomly selected in order to confirm their identity and were sequenced using an ABI3130xl genetic analyzer with Big Dye ver3.1 following the manufacturer’s instruction (Applied Biosystems). The obtained sequences were assembled and analyzed with Sequencer ver 4.10.1 (Gene Codes). The 16S rRNA genes sequences obtained in this study have been deposited in the DDBJ/EMBL/GenBank database under the following accession number: AB983194. The 16S rRNA gene sequences of related strains retrieved from the DNA Data Bank of Japan were aligned using the CLUSTAL X 2.0.12 multiple alignment accessory application [30–33]. A phylogenetic tree was reconstructed using the neighbor-joining (NJ) method and evaluated by bootstrap sampling [34,35].

### Extraction and purification of magnetic nanoparticles

Magnetic nanoparticles were extracted from the cells at a stationary phase and purified removing cell debris by a modified method previously used [36]. The cells were harvested by centrifugation under 5,000 × *g* at 4°C for 30 min and the supernatant was completely removed. The pelleted cells were washed three times with 10 mM HEPES buffer (pH 7.4) containing 1 mM ethylenediamine tetra-acetic acid (EDTA) to remove thoroughly metallic ions including samarium ions from the surface of the cells. The cells washed with EDTA were resuspended in 1 M NaOH and boiled for 20 min. Magnetic nanoparticles were collected using a neodymium-boron magnets (ϕ10 × 10 mm), washed with sterile distilled water at least three times using a sonicator (28 kHz, 10 min) (W-113, HONDA) and then stored at 4°C in sterile distilled water for later structural, elemental and magnetic analyses.

### Transmission electron microscopic (TEM) observation of cells

For transmission electron microscopic (TEM) observations, cells at a stationary phase were placed on a TEM grid (200 mesh Cu Formvar/carbon-coated grid, JEOL) and air-dried at room temperature. The grid was rinsed three times with sterile distilled water and then the cells were observed by a TEM (JEM-2100, JEOL) with an accelerating voltage of 200 kV. The number of magnetic nanoparticles in each cell was counted targeting at 100 individual cells [37]. The size of magnetic nanoparticles was counted measuring at least 1000 purified magnetic nanoparticles from several TEM micrographs.

### TEM and Energy-dispersive X-ray spectrometric (EDS) analyses of magnetic nanoparticles

The purified magnetic nanoparticles were mounted on a TEM grid and air-dried. High-resolution scanning TEM (HRSTEM)-EDS analysis was performed using a TEM (JEM-2200FS, JEOL) operated at 200 kV with accumulation time for approximately 60 s. High-resolution EDS elemental mapping analysis was conducted using a TEM (JEM-ARM200F with a Schottky gun, JEOL) equipped with a probe aberration corrector at an acceleration voltage of 200 kV.

### X-ray photoelectron spectroscopic (XPS) analysis of magnetic nanoparticles

XPS analysis was carried out using an X-ray photoelectron spectrometer (Kratos AXIS-HSi, Shimadzu) with a monochromatic Al Kα (1486.6 eV) radiation source. The binding energy was calibrated with the measurement of the adventitious C 1*s* (284.6 eV) signal, which was caused by remaining hydrocarbon.

### Measurement of the magnetic properties of magnetic nanoparticles extracted from and contained in cells

The purified magnetic nanoparticles were dried in a vacuum chamber and powder capsules (P125E, Quantum Design), into which the samples had been introduced, were mounted in brass sample holders for the measurement of the magnetic properties by a superconducting quantum interference device (SQUID) magnetometer (MPMS3, Quantum Design) at 4 and 300 K. The zero field cooling (ZFC) and field cooling (FC) magnetization curves of the magnetic particles were obtained in the temperature range from 4 to 300 K in an applied field of 50 Oe (= 50 × 10^3^/4π A m^-1^). The mass magnetization–magnetic field curves were also obtained at 4 and 300 K. The magnetic properties of magnetic nanoparticles in intact cells were measured by SQUID after the cells had been freeze dried.

### Raman spectroscopic analysis of magnetic nanoparticles

Micro-Raman spectroscopy was performed using a Raman microscope (HR-800UV, Horiba-Jobin Yvon LabRAM, Horiba) at room temperature. A laser wavelength of 325 nm was used as the excitation source.

### X-ray powder diffraction (XRD) analysis of magnetic nanoparticles

The crystalline structures of the nanoparticles were identified by X-ray diffractometry (SmartLab, Rigaku), with a Cu radiation source (Kα = 1.5418 Å) at 45 kV and 200 mA. For grazing incidence XRD measurement, we used a fixed incident angle of 0.01°. The scan angle was changed between 5° and 120° with a scan size of 0.2° and a scan time of 0.5 s per 0.2°.

## Results

### Growth of strain RSS-1 and synthesis of magnetic nanoparticles in strain RSS-1

**Fig 1:**
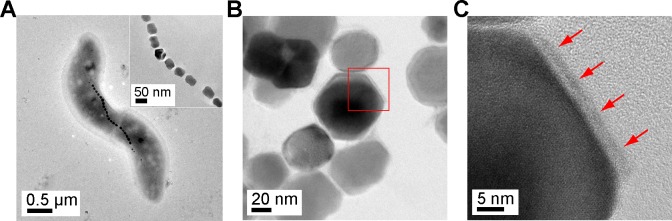
TEM images of strain RSS-1, magnetosomes and extracted magnetic nanoparticles. (a) TEM image of a strain RSS-1 cell grown in the medium containing 250 μM Fe-q:250 μM Sm-q. (b) HRTEM image of magnetic nanoparticles extracted from strain RSS-1 grown in the presence of 250 μM Fe-q:250 μM Sm-q. (c) Magnified image of a magnetic nanoparticle indicated by a red square in image (b). Red arrows indicate a surface layer covering the core magnetic nanoparticle.

We isolated strain RSS-1 cells from sediment and grew them in semi-solid agar containing 250 μM Fe quinate (Fe-q) and 250 μM Sm quinate (Sm-q). We confirmed that magnetosomes were formed in the cells (see Fig 1A), noting that no magnetosomes were initially contained in the cells in a preculture medium. A TEM image shows that the cells were spirilla in morphology with a width and length of 0.6 ± 0.1 and 3.2 ± 0.7 μm. The surface of each magnetic particle synthesized by strain RSS-1 in the presence of 250 μM Fe-q and 250 μM Sm-q was covered with a thin layer (Fig 1B and 1C), which will be analyzed and discussed in more detail later. We also confirmed that the surface of each magnetic particles synthesized by strain RSS-1 in the presence of only 250 μM Fe-q was not covered with any other layer including lipid membranes (S1 Fig).

In order to confirm axenic cultivation of strain RSS-1, we measured the sequences of the 16S rRNA gene of 7 clones of the strain RSS-1 cultures and found that the clones showed more than 99.7% similarity with each other. The neighbor-joining tree showed that strain RSS-1 was a member of the family *Rhodospirillaceae* of *Alphaproteobacteria* and was most closely related to *Magnetospirillum magneticum* (see S2 Fig in the Supplementary material). Similarity search performed using the National Center for Biotechnology Data Basic Local Alignment Tool (NCBI BLAST) showed that the 16S rRNA gene sequence of strain RSS-1 was most closely related to *M*. *magneticum* AMB-1, the mutual genes having shown 99.9% similarity.

**Fig 2:**
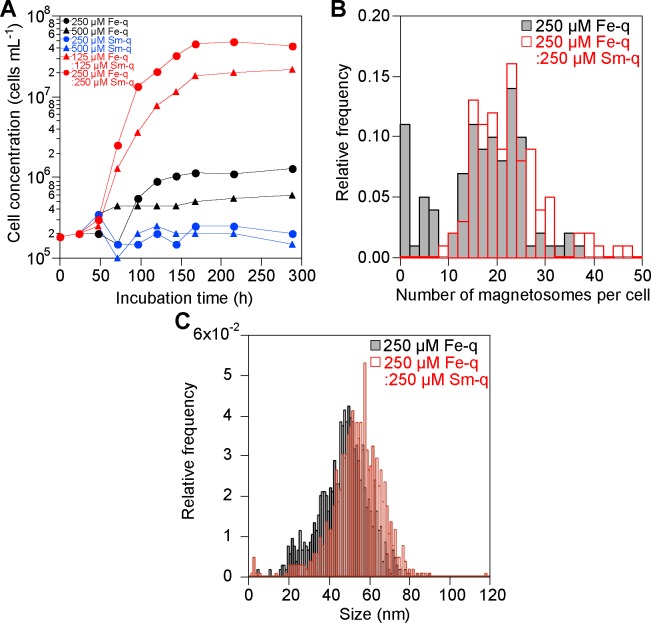
Growth curves of strain RSS-1, the number of magnetic nanoparticles synthesized in each cell and the size distribution of Fe_3_O_4_ NPs and Fe_3_O_4_@Sm_2_O_3_ core-shell NPs. (a) Growth of strain RSS-1 in the medium containing iron and/or samarium quinate. (b) Distribution of the number of magnetic nanoparticles in each cell. (c) Distribution of the size of Fe_3_O_4_ NPs and Fe_3_O_4_@Sm_2_O_3_ core-shell NPs.

To investigate the effect of samarium and iron on the growth of strain RSS-1 and the formation of magnetosomes, RSS-1 cells were grown in the presence of Fe quinate and/or Sm quinate. The growth curves of strain RSS-1 in the liquid medium containing 250 μM Fe-q, 500 μM Fe-q, 250 μM Sm-q, 500 μM Sm-q, 125 μM Fe-q:125 μM Sm-q and 250 μM Fe-q:250 μM Sm-q under an O_2_ (1%)-N_2_ (99%) atmosphere at 28°C in the dark are shown in Fig 2A. The final cell concentration was 1.3 × 10^6^, 6.0 × 10^5^, 1.5 × 10^5^, 2.0 × 10^5^, 2.2 × 10^7^ and 4.3 × 10^7^ cells mL^-1^, respectively, in the presence of 250 μM Fe-q, 500 μM Fe-q, 250 μM Sm-q, 500 μM Sm-q, 125 μM Fe-q:125 μM Sm-q and 250 μM Fe-q:250 μM Sm-q. Thus, strain RSS-1 grew most rapidly in the presence of 250 μM Fe-q:250 μM Sm-q. Note that we carried out 50 experiments on the growth of strain RSS-1 in the presence of 250 μM Fe-q:250 μM Sm-q and confirmed that there was no significant difference in the final cell concentration. Furthermore, the number of magnetic nanoparticles per cell grown in the presence of 250 μM Fe-q:250 μM Sm-q for 11 days was 20.9 ± 7.2, whereas that in the presence of only 250 μM Fe-q was 15.3 ± 8.6 (see Fig 2B). In other words, there was approximately 37% increase in the average number of magnetic nanoparticles in each cell in the case of 250 μM Fe-q:250 μM Sm-q. Judging by the present result and a previous report; that is, the size and number of magnetic nanoparticles in *M*. *magnetotacticum* MS-1 were increased in the presence of zinc and nickel salt [38], it is supposed that the number of magnetic nanoparticles in strain RSS-1 might have been increased by samarium added to the culture medium. The size of Fe_3_O_4_@Sm_2_O_3_ core-shell NPs was larger than that of Fe_3_O_4_ NPs by approximately 14%, noting that 52.8 ± 12.2 nm in the former case, while 46.2 ± 12.4 nm in the latter (see Fig 2C). The thickness of Sm_2_O_3_ in the Fe_3_O_4_@Sm_2_O_3_ core-shell NPs was therefore approximately 3 nm (see also Fig 1C).

### XPS analysis of magnetic nanoparticles

**Fig 3:**
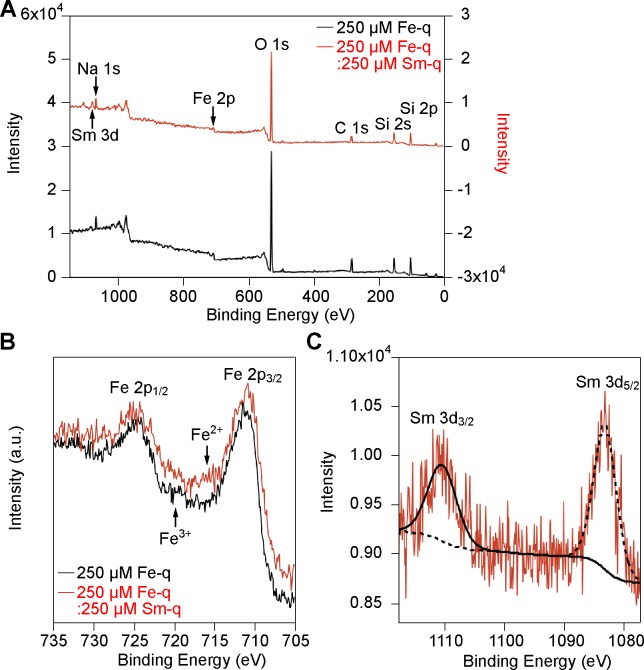
XPS analysis of magnetic nanoparticles. (a) Full XPS spectra of the magnetic nanoparticles synthesized by strain RSS-1 grown in the presence of 250 μM Fe-q and 250 μM Fe-q:250 μM Sm-q. A monochromatic Al Kα radiation source was used. (b) Fe 2*p* spectra of the magnetite nanoparticles synthesized by strain RSS-1 grown in the presence of 250 μM Fe-q and 250 μM Fe-q:250 μM Sm-q. (c) Sm 3*d* spectrum of the magnetite nanoparticles synthesized by strain RSS-1 grown in the presence of 250 μM Fe-q:250 μM Sm-q.

XPS analysis was carried out to verify the presence of Fe, Sm, and O and to determine the oxidation states of the metal ions (Fig 3). Fig 3A shows the presence of Fe, Sm, and O in/on the magnetic nanoparticles extracted from strain RSS-1. The magnetic nanoparticles were composed of Fe_3_O_4_ irrespective of the presence of samarium, confirmed by the Fe 2*p*_1/2_ and Fe 2*p*_3/2_ peaks at around 724 and 710 eV (Fig 3B), which coincided with those of Fe_3_O_4_ [39]. Comparing the binding energies (3*d*_3/2_ and 3*d*_5/2_) of Sm (III) present in the magnetite crystals; that is, 1110.2 and 1083.0 eV, with those of standard Sm_2_O_3_; i.e., 1110.4 and 1083.2 eV, it can be concluded that samarium is present in the +3 oxidation state in the nanoparticles (Fig 3C).

### EDS analysis of magnetic nanoparticles

**Fig 4:**
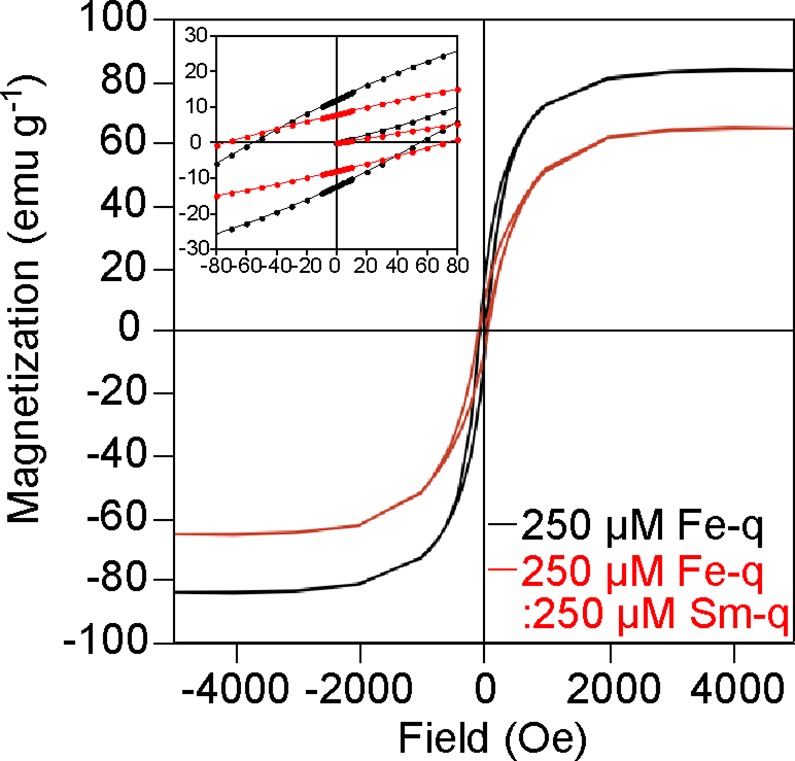
HRSTEM image and STEM-EDS elemental maps of magnetite nanoparticles. STEM-EDS elemental maps corresponding to Fe, O and Sm, and overlay of the three elements are shown.

We performed HRSTEM-EDS elemental mappings of the nanoparticles (see Fig 4). It is clearly shown that samarium was present on the magnetic nanoparticles extracted from strain RSS-1 grown in the presence of 250 μM Fe-q:250 μM Sm-q for 11 days. Note that metallic ions including samarium ones were completely removed from the surface of the cells before the extraction of magnetic nanoparticles and therefore there was hardly any chance of the inclusion of samarium onto/into magnetic nanoparticles during the extraction process. S3 Fig shows spot EDS spectra obtained from peripheral and central areas of magnetic nanoparticles. Sm is clearly detected from both areas of the particle. Note that EDS signals corresponding to Sm were still clearly detected from magnetosomes in the cells (see S4 Fig). Phosphorus (P), sulfur (S), iron (Fe), oxygen (O) and samarium (Sm) were detected near the edge of a particles in a cell, which shows that a particle composed of Fe, O and Sm is captured in a membrane containing P and S, assuming that P and S are originated from a phospholipid bilayer and some protein (S4 Fig), whereas neither P nor S were detected from the particles extracted from the cells (see S3 Fig).

### Magnetic properties of Fe_3_O_4_@Sm_2_O_3_ core-shell NPs and Fe_3_O_4_ NPs

**Fig 5:**
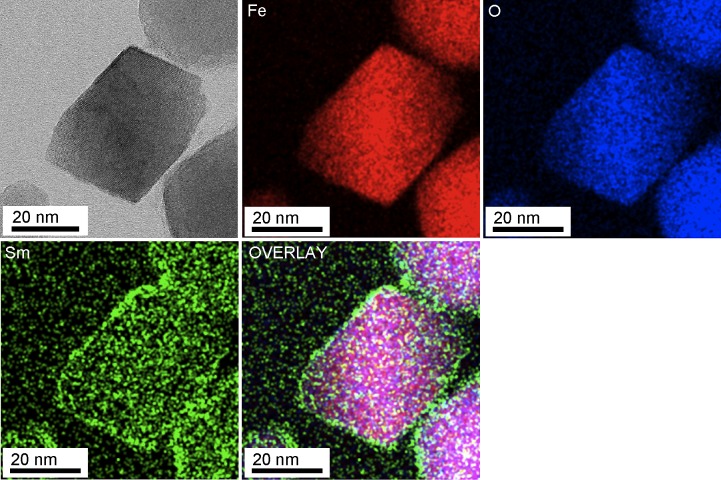
Magnetization–magnetic field curves of Fe_3_O_4_ NPs and Fe_3_O_4_@Sm_2_O_3_ core-shell NPs. Magnetic hysteresis loops of Fe_3_O_4_ NPs and Fe_3_O_4_@Sm_2_O_3_ core-shell NPs were measured at 300 K.

**Fig 6:**
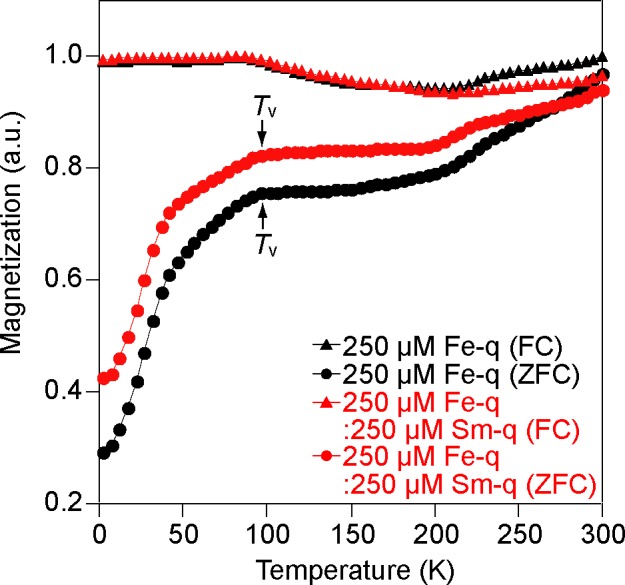
ZFC and FC magnetization curves. The curves are normalized by the maximum values of the saturation magnetization. Field-cooling (FC) and zero-field-cooling (ZFC) measurements of the magnetization of magnetite nanoparticles synthesized by strain RSS-1 grown in the presence of 250 μM Fe-q and 250 μM Fe-q:250 μM Sm-q are shown. Arrows show the Verwey transition temperature (*T*_v_).

The magnetic properties of Fe_3_O_4_@Sm_2_O_3_ core-shell NPs and Fe_3_O_4_ NPs were measured at 4 and 300 K using a SQUID magnetometer. The mass magnetization–magnetic field curves are shown in Fig 5 and the magnetic properties of Fe_3_O_4_@Sm_2_O_3_ core-shell NPs and Fe_3_O_4_ NPs are summarized in Table 1. The saturation mass magnetization (*m*_s_), residual mass magnetization (*m*_r_) and coercivity (*H*_c_) of Fe_3_O_4_@Sm_2_O_3_ core-shell NPs measured at 300 K were 61.4 emu g^-1^ (= 7.71 × 10^−5^ Wb m kg^-1^), 7.3 emu g^-1^ (= 9.17 × 10^−6^ Wb m kg^-1^) and 65.1 Oe (= 5.18 × 10^3^ A m^-1^), whereas those of Fe_3_O_4_ NPs were 88.5 emu g^-1^ (= 1.11 × 10^−4^ Wb m kg^-1^), 13.9 emu g^-1^ (= 1.75 × 10^−5^ Wb m kg^-1^) and 54.3 Oe (= 4.32 × 10^3^ A m^-1^) (Fig 5 and Table 1). The saturation mass magnetization and residual mass magnetization of Fe_3_O_4_@Sm_2_O_3_ core-shell NPs decreased by approximately 31 and 48% compared to those of Fe_3_O_4_ NPs, while the coercivity of Fe_3_O_4_@Sm_2_O_3_ core-shell NPs increased by approximately 17%. *m*_s_, *m*_r_ and *H*_c_ of Fe_3_O_4_@Sm_2_O_3_ core-shell NPs measured at 4 K were 72.1 emu g^-1^ (= 9.06 × 10^−5^ Wb m kg^-1^), 20.0 emu g^-1^ (= 2.51 × 10^−5^ Wb m kg^-1^) and 272 Oe (= 2.17 × 10^4^ A m^-1^), whereas those of Fe_3_O_4_ NPs was 91.1 emu g^-1^ (= 1.14 × 10^−4^ Wb m kg^-1^), 29.0 emu g^-1^ (= 4.96 × 10^−5^ Wb m kg^-1^) and 232 Oe (= 1.85 × 10^4^ A m^-1^) (S5 Fig). The saturation mass magnetization and residual mass magnetization of Fe_3_O_4_@Sm_2_O_3_ core-shell NPs decreased by approximately 21 and 31%, while the coercivity increased by approximately 17%. Considering that the thickness of Sm_2_O_3_ in the Fe_3_O_4_@Sm_2_O_3_ core-shell NPs was approximately 3 nm (Figs 1C, 2C and 4) and that the mass magnetization of Sm_2_O_3_ is extremely low (see S7 Fig), it is supposed that the decrease in the saturation mass magnetization of the Fe_3_O_4_@Sm_2_O_3_ core-shell NPs was simply caused by the mass increase by Sm_2_O_3_. There is a report that the coercivity of magnetic nanoparticles in *M*. *magneticum* AMB-1 was approximately 270 Oe at 300 K [15], which is greater than that measured in the present study, where magnetic particles were extracted from the cells. We therefore measured the magnetic properties of magnetosomes in freeze-dried cells of strain RSS-1 cultivated in the presence of samarium, according to which the coercivity was 175.9 Oe (= 1.40 × 10^4^ A m^-1^), whereas that in the absence of samarium was 185.8 Oe (= 1.48 × 10^4^ A m^-1^) (see S6 Fig), which were slightly lower than the previous report [15]. The magnetic properties of particles change depending on the configurations of particles and the temperature. The theoretical value of coercivity of a non-interacting, isometric, single-domain magnetite is approximately 200 Oe, whereas the coercivity of clusters formed via the interaction between magnetite nanoparticles is lowered [40]. The coercivity of magnetite nanoparticles in a chain, in which case the particles are aligned along their magnetic easy axes in the cells, increases due to the shape anisotropy [40–44]. It is well known that the Verwey transition is characterized by the change of the magnetocrystalline anisotropy due to the crystallographic transition of magnetite from cubic to monoclinic [45,46]. Fig 6 shows the ZFC and FC curves of Fe_3_O_4_@Sm_2_O_3_ core-shell NPs and Fe_3_O_4_ NPs, which indicate that the Verwey transition temperature (*T*_v_) of both Fe_3_O_4_@Sm_2_O_3_ core-shell NPs and Fe_3_O_4_ NPs was approximately 100 K. In other words, there was no significant difference in the Verwey transition temperature between Fe_3_O_4_@Sm_2_O_3_ core-shell NPs and Fe_3_O_4_ NPs, from which it is supposed that the core particles consist of pure magnetite.

**Table 1:** Magnetic properties of Fe_3_O_4_@Sm_2_O_3_ core-shell NPs and Fe_3_O_4_ NPs.

	Fe_3_O_4_@Sm_2_O_3_ core-shell NPs*	Fe_3_O_4_ NPs*
*m*_s_^300K^ (emu g^-1^)	61.4 ± 12.4	88.5 ± 4.1
*m*_r_^300K^ (emu g^-1^)	7.3 ± 0.6	13.9 ± 2.3
*H*_c_^300K^ (Oe)	65.1 ± 13.4	54.3 ± 0.9

* The *m*_*s*_, *m*_*r*_ and *H*_*c*_ values were obtained from three independent experiments.

### Structures of Fe_3_O_4_@Sm_2_O_3_ core-shell NPs and Fe_3_O_4_ NPs

Magnetosomes are usually composed of Fe_3_O_4_, but they can be oxidized to form γ-Fe_2_O_3_ [7]. It is also possible that α-Fe_2_O_3_ is present on the surface of immature magnetosomes [47]. To investigate the crystal structures of the present Fe_3_O_4_@Sm_2_O_3_ core-shell NPs, we performed Raman spectroscopic and XRD analyses (S7 Fig). The Raman spectra of both Fe_3_O_4_@Sm_2_O_3_ core-shell NPs and Fe_3_O_4_ NPs with an excitation wavelength of 325 nm are identical, having main peaks at around 344, 510 and 728 cm^-1^, but are completely different from those of FeO, α-Fe_2_O_3_ and γ-Fe_2_O_3_ (see S7A Fig). S7B Fig shows the XRD patterns of both Fe_3_O_4_@Sm_2_O_3_ core-shell NPs and Fe_3_O_4_ NPs, which confirms that both nanoparticles have a cubic spinel structure of magnetite (Fe_3_O_4_), referring to the Inorganic Crystal Structural Database (ICSD) 159971. It is supposed that the structure of the surface layers composed of Sm_2_O_3_ was amorphous without any clear peaks of crystallized Sm_2_O_3_ in the XRD pattern [48].

## Discussion

It had been assumed that lanthanides can be bound to the cell surface of bacteria, but they cannot be transported into the cytoplasm of bacteria [21,49]. However, it was shown that lanthanum and terbium accumulate in the periplasmic space of *Escherichia coli* [23], which suggests that lanthanides may be transported into the periplasmic space of MTB, knowing that all of MTB possess a cell wall structural characteristic of gram-negative bacteria [7].

It was hypothesized that there may be several steps in the process of the formation of magnetosomes in *Magnetospirillum* species; that is, (a) the formation of vesicles via invagination of the inner membrane, (b) uptake of extracellular iron into the cell, (c) transport of iron into the membrane vesicles, and (d) nucleation and growth of magnetite crystals in the vesicles [50,51]. Thus, there is a possibility that lanthanides may be transported into the magnetosome membrane vesicles passing through the periplasmic space. We showed that the growth rate of strain RSS-1 and the number of magnetic nanoparticles synthesized in each cell were increased by adding both samarium and iron to the culture media, which suggests that the addition of some transition metals with iron to the culture media may in general encourage the growth of MTB and the formation of magnetosomes via active uptake of iron rather than stochastic transport of iron into the periplasmic space. In the case of cobalt doped magnetosomes, Co was found to be localized in the surface layer of the magnetite crystals [15], which indicates that Fe was a seed material in the initial phases of the formation of magnetite crystals. The present study showed that core magnetite nanoparticles were surrounded by samarium oxide (see Fig 4 and S4 Fig). It is therefore supposed that strain RSS-1 might have incorporated samarium into magnetosomes in a terminal phase of the formation of magnetite crystals. It may be possible to understand the formation mechanism of core-shell particles in the cells during the biomineralisation process by carrying out both structural and elemental observation and analyses as a function of time.

The possibility of MTB being used as biosorbents has been investigated since MTB can be easily recovered using magnets [52–56]. Several methods of separating rare earth elements from solution; e.g., the solvent extraction method, have been developed using some chemical treatments. However, a large amount of organic solvent is used in such treatment, which should be avoided for environmental safety. It was clearly shown in the present study that strain RSS-1 grew rapidly in the presence of 250 μΜ Fe quinate and 250 μΜ Sm quinate (Fig 2A). Moreover, the number of Fe_3_O_4_@Sm_2_O_3_ core-shell NPs in the presence of 250 μΜ Fe quinate and 250 μΜ Sm quinate was larger than that in the presence of only 250 μΜ Fe quinate (Fig 2B). Therefore, we believe that strain RSS-1 may well be utilized for magnetic recovery of samarium.

The properties of hybrid nanomaterials such as core-shell NPs, core-shell nanorods and nanonecklaces are generally different from those of single component nanomaterials [57–59]. Fe_3_O_4_@SiO_2_-Sm_2_O_3_ core-shell NPs are expected to be used as a magnetic diagnostic material thanks to Sm_2_O_3_ [60]. We hope that the Fe_3_O_4_@Sm_2_O_3_ core-shell NPs presently synthesized by strain RSS-1 will be used in a wide range of fields including electronic, magnetic and biomedical studies, based on the outstanding features of the hybrid particles; i.e., the core Fe_3_O_4_ NPs will be prevented from oxidation and corrosion and the specific properties of Sm_2_O_3_ covering the core particles will still be maintained even after extraction. We will be investigating the effect of other transition metals added to the culture media on the growth of MTB and the formation and structures of magnetosomes so that some universal features in the formation and structures of magnetosomes may be revealed, which we suppose may make a great contribution to the development of environmentally friendly nanomaterials synthetic technologies.

In summary, we successfully grew *Magnetospirillum magneticum* strain RSS-1 and found that strain RSS-1 grew most rapidly and, what is more, the number of magnetic nanoparticles per cell increased in the presence of both Fe and Sm. Each core particle composed of magnetite was covered with a thin layer of samarium oxide. The present results show some possibilities of magnetic recovery of transition metals and synthesis of some novel structures composed of magnetic particles and transition metals utilizing Magnetotactic bacteria.

## Supporting Information

S1 Fig TEM and HRTEM images of magnetic nanoparticles synthesized by strain RSS-1 in the absence of samarium. Magnetic nanoparticles were extracted from strain RSS-1 grown in the presence of only 250 μM Fe-q.
(TIF)

S2 Fig Neighbor-joining phylogenetic tree. Neighbor-joining phylogenetic tree constructed based on the 16S rRNA gene sequences. The position of strain RSS-1 and some other related *Magnetospirillum* are represented. Bootstrap values per 1,000 replicates are indicated. The GeneBank accession numbers are shown in parentheses. Bar, 0.01 changes per nucleotide position.
(TIF)

S3 Fig STEM-EDS analysis of a magnetic nanoparticle extracted from strain RSS-1. STEM-EDS spot analysis of peripheral (i) and central (ii) areas of a magnetic nanoparticle indicated by asterisks is shown.
(TIF)

S4 Fig STEM-EDS analysis of a magnetic nanoparticle in a cell. STEM-EDS spot analysis of a peripheral area of a magnetic nanoparticle indicated by an asterisk is shown. Cu signals are due to the TEM grid used, whereas the Cr signal is attributed to Cr plating on the sample holder.
(TIF)

S5 Fig Magnetization–magnetic field curves of Fe_3_O_4_ NPs and Fe_3_O_4_@Sm_2_O_3_ core-shell NPs measured at 4 K. The black and red circles represent Fe_3_O_4_ NPs and Fe_3_O_4_@Sm_2_O_3_ core-shell NPs.
(TIF)

S6 Fig Magnetization–magnetic field curves of magnetosomes in freeze-dried cells of strain RSS-1 cultivated in the culture medium in the absence and in the presence of samarium. The black and red lines represent the mass magnetization in the absence and in the presence of samarium. The mass magnetization was measured at 300 K.
(TIF)

S7 Fig Raman spectroscopic and XRD analyses of Fe_3_O_4_@Sm_2_O_3_ core-shell NPs and Fe_3_O_4_ NPs. (a) Raman spectrum of the magnetite crystals with an excitation wavelength of 325 nm. The spectra corresponding to FeO, Fe_3_O_4_, α-Fe_2_O_3_ and γ-Fe_2_O_3_ are represented as references. (b) XRD of Fe_3_O_4_@Sm_2_O_3_ core-shell NPs and Fe_3_O_4_ NPs.
(TIF)

## References

[1] Blakemore RP, Maratea D, Wolfe RS (1979) Isolation pure culture of a freshwater magnetic spirillum in chemically defined medium. J. Bacteriol. 140: 720–729. 500569 10.1128/jb.140.2.720-729.1979 PMC216702

[2] Bazylinskii DA, Frankel RB, Heywood BR, Mann S, King JW, Donaghay PL, et al (1995) Controlled biomineralization of magnetite (Fe_3_O_4_) and greigite (Fe_3_S_4_) in a magnetotactic bacterium. Appl. Environ. Microbiol. 61: 3232–3239. 16535116 10.1128/aem.61.9.3232-3239.1995 PMC1388570

[3] Sparks NHC, Courtaux L, Mann S, Board RG (1986) Magnetotactic bacteria are widely distributed in sediments in the U.K. FEMS Microbiol. Lett. 37: 305–308.

[4] Mann S, Sparks NHC, Board RG (1990) Magnetotactic Bacteria: Microbiology, Biomineralization, Palaeomagnetism and Biotechnology. Adv. Microbiol. Physiol. 31: 125. 2124779 10.1016/s0065-2911(08)60121-6

[5] Bazylinski DA, Garratt-Reed AJ, Frankel RB (1994) Electron microscope study of magnetosomes in two cultured vibrioid magnetotactic bacteria. Microsc. Res. Technol. 27: 389–401. 10.1002/jemt.1070270505 8018991

[6] Heyen U, Schüler D (2003) Growth and magnetosome formation by microaerophilic *Magnetospirillum* strains in oxygen-controlled fermentor. Appl. Microbiol. Biotechnol. 61: 536–544. 10.1007/s00253-002-1219-x 12764570

[7] Bazylinski DA, Frankel R (2004) Magnetosome formation in prokaryotes. Nat. Rev. Microbiol. 2: 217–230. 10.1038/nrmicro842 15083157

[8] Xiang L, Wei J, Jianbo S, Guili W, Feng G, Ying L (2007) Purified and sterilized magnetosomes from *Magnetospirillum gryphiswaldense* MSR-1 were not toxic to mouse fibroblasts in vitro. Lett. Appl. Microbiol. 45: 75–81. 10.1111/j.1472-765X.2007.02143.x 17594464

[9] Sun J, Tang T, Duan J, Xu PX, Wang Z, Zhang Y, et al (2010) Biocompatibility of bacterial magnetosomes: acute toxicity, immunotoxicity and cytotoxicity. Nanotoxicology 4: 271–283. 10.3109/17435391003690531 20795909

[10] Duguet E, Vasseur S, Mornet S, Devoisselle JM (2006) Magnetic nanoparticles and their applications in medicine. Nanomedicine 1: 157–168. 10.2217/17435889.1.2.157 17716105

[11] Keim CN, Lins U, Farina M (2009) Manganese in biogenic magnetite crystals from magnetotactic bacteria. FEMS Microbiol. Lett. 292: 250–253. 10.1111/j.1574-6968.2009.01499.x 19187208

[12] Towe KM, Moench TT (1981) Electron-optical characterization of bacterial magnetite. Earth Planet. Sci. Lett. 52: 213–220.

[13] Bazylinski DA, Garrattreed AJ, Abedi A, Frankel RB (1993) Copper association with iron sulfide magnetosomes in a magnetotactic bacterium. Arch. Microbiol. 160: 35–42.

[14] Posfai M, Buseck PR, Bazylinski DA, Frankel RB (1998) Iron sulfides from magnetotactic bacteria: structure, composition, and phase transitions. Am. Mineral. 83: 1469–1481.

[15] Staniland S, Williams W, Telling N, Van Der Laan G, Harrison A, Ward B (2008) Controlled cobalt doping of magnetosomes *in vivo*. Nat. Nanotechnol. 3: 158–162. 10.1038/nnano.2008.35 18654488

[16] Li J, Menguy N, Arrio M-A, Liang M (2016) Controlled cobalt doping in the spinel structure of magnetosome magnetite: New evidences from element- and site-specific X-ray magnetic circular dichroism analyses. J. R. Soc. Interface 13: 20160355 10.1098/rsif.2016.0355 27512138 PMC5014062

[17] Tanaka M, Brown R, Hondow N, Arakaki A, Matsunaga T, Staniland S (2012) High levels of Cu, Mn and Co doped into nanomagnetic magnetosomes through optimized biomineralisation. J. Mater. Chem. 22: 11919–11921.

[18] Prozorov T, Perez-Gonzalez T, Valverde-Tercedor C, Jimenez-Lopez C, Yebra-Rodriguez A, Körnig A, et al (2014) Manganese incorporation into the magnetosome magnetite: magnetic signature of doping. Eur. J. Mineral. 26: 457–471.

[19] Liang X, Wang X, Zhuang J, Chen Y, Wang D, Li Y (2006) Synthesis of nearly monodisperse iron oxide and oxyhydroxide nanocrystals. Adv. Func. Mater. 16: 1805–1813.

[20] De Silva CR, Smith S, Shim I, Pyun J, Gutu T, Jiao J, et al (2009) Lanthanide (III)-doped magnetite nanoparticles. J. Am. Chem. Soc. 131: 6336–6337. 10.1021/ja9014277 19368388

[21] Takahashi Y, Châtellier X, Hattori KH, Kato K, Fortin D (2005) Adsorption of rare earth elements onto bacterial cell walls and its implication fro REE sorption onto natural microbial mats. Chem. Geol. 219: 53–67.

[22] Ngwenya BT, Magennis M, Olive V, Mosselmans JF, Ellam RM (2010) Discrete site surface complexation constants for lanthanide adsorption to bacteria as determined by experiments and linear free energy relationships. *Environ*. *Sci*. *Technol*. 44: 650–656. 10.1021/es9014234 20000843

[23] Bayer ME, Bayer MH (1991) Lanthanide accumulation in the periplasmic space of *Escherichia coli* B. J. Bacteriol. 173: 141–149. 1987113 10.1128/jb.173.1.141-149.1991 PMC207167

[24] Tsuruta T (2006) Selective accumulation of light or heavy rare earth elements using gram-positive bacteria. *Colloids Surf*. *B*. *Biointerfaces* 52: 117–22. 16797944 10.1016/j.colsurfb.2006.04.014

[25] Sorokin PP (1979) Contribution of IBM to laser science. IBM J. Res. Dev. 23: 476–489.

[26] Hammond CR (2000) *The elements*. *Handbook of chemistry and physics*, 81th ed (Boca Raton: CRC press)

[27] Cotton S (2006) Lanthanide and Actinide Chemistry (West Sussex: John Wiley and Sons)

[28] Jogler C, Lin W, Meyerdierks A, Kube M, Katzmann E, Flies C, et al (2009) Toward cloning of the magnetotactic metagenome: identification of magnetosome island gene clusters in uncultivated magnetotactic bacteria from different aquatic sediments. Appl. Environ. Microbiol. 75: 3972–3979. 10.1128/AEM.02701-08 19395570 PMC2698345

[29] Lane DJ (1991) *16S/23S rRNA sequencing*: *Nucleic acid techniques in bacterial systematics*, Stackebrandt E and Goodfellow M, eds (New York: John Wiley and Sons) pp.115–175.

[30] Miyazaki S, Sugawara H, Gojobori T, Tateno Y (2003) DNA Data Bank of Japan (DDBJ) in XML. Nucleic. Acids Res. 31: 13–16. 12519938 10.1093/nar/gkg088 PMC165535

[31] Lipman DJ, Pearson WR (1985) Rapid and sensitive protein similarity searches. Science 227: 1435–1441. 2983426 10.1126/science.2983426

[32] Pearson WR, Lipman DJ (1988) Improved tools for biological sequence comparison. Proc. Natl. Acad. Sci. USA 85: 2444–2448. 3162770 10.1073/pnas.85.8.2444 PMC280013

[33] Larkin MA, Blackshields G, Brown NP, Chenna R, McGettigan PA, McWilliam H, et al (2007) CLUSTAL W and CLUSTAL X version 2.0. Bioinformatics 23: 2947–2948. 10.1093/bioinformatics/btm404 17846036

[34] Saitou N, Nei M (1987) The neighbour-joining method: a new method for reconstructing phylogenetic trees. Mol. Biol. Evol. 4: 406–425. 3447015 10.1093/oxfordjournals.molbev.a040454

[35] Felsenstein J (1985) Confidence limits on phylogenies: an approach using the bootstrap. Evolution 39: 783–791. 28561359 10.1111/j.1558-5646.1985.tb00420.x

[36] Yang CD, Takeyama H, Tanaka T, Matsunaga T (2001) Effects of growth medium composition, iron sources and atmospheric oxygen concentrations on production of luciferase-bacterial magnetic particle complex by a recombinant *Magnetospirillum magneticum* AMB-1. Enzyme Microb. Technol. 29: 13–19. 11427230 10.1016/s0141-0229(01)00343-x

[37] Shimoshige H, Kobayashi H, Mizuki T, Nagaoka Y, Inoue A, Maekawa T (2015) Effect of polyethylene glycol on the formation of magnetic nanoparticles synthesized by *Magnetospirillum magnetotacticum* MS-1. PLoS One 10: e0127481 10.1371/journal.pone.0127481 25993286 PMC4439050

[38] Kundu S, Kale AA, Anpurkar AG, Kulkarni GR, Ogale SB (2009) On the change in bacterial size and magnetosome features for *Magnetospirillum magnetotacticum* (MS-1) under high concentrations of zinc and nickel. Biomaterials 30: 4211–4218. 10.1016/j.biomaterials.2009.04.039 19500838

[39] Yamashita T, Hayes P (2008) Analysis of XPS spectra of Fe^2+^ and Fe^3+^ ions in oxide metarials. Appl. Suf. Sci. 254: 2441–2449.

[40] Charilaou M, Winklhofer M, Gehring AU (2016) Simulation of ferromagnetic resonance spectra of linear chains of magnetite nanocrystals. J. Appl. Phys. 109:093903.

[41] Moskowitz BM, Frankel RB, Bazylinski DA (1993) Rock magnetic criteria for the detection of biogenic magnetite. Earth Planet. Sci. Lett. 120: 283–300.

[42] Li J, Wu W, Liu Q, Pan Y (2012) Magnetic anisotropy, magnetostatic interactions and identification of magnetofossils. Geochem. Geophys. 13: Q10Z51.

[43] Dunin-Borkowski RE, McCartney MR, Frankel RB, Bazylinski DA, Posfai M, Buseck PR (1998) Magnetic microstructure of magnetotactic bacteria by electron holography. Science 282: 1868–1870. 9836632 10.1126/science.282.5395.1868

[44] Li J, Ge K, Pan Y, Williams W, Liu Q, Qin H (2013) A strong angular dependence of magnetic properties of magnetosome chains: Implications for rock magnetism and paleomagnetism. Geochem. Geophys. 14: 3887–3907.

[45] Gehring AU, Fischer H, Charilaou M, García-Rubio I (2011) Magnetic anisotropy and Verwey transition of magnetosome chains in *Magnetospirillum gryphiswaldense*. Geophys. J. Int. 187: 1215–1221.

[46] Pan YX, Pertersen N, Winklhofer M, Davila AF, Liu Q, Frederichs T, et al (2005) Rock magnetic properties of uncultured magnetotactic bacteria. Earth Planet. Sci. Lett. 237 (3–4): 311–325.

[47] Komeili A, Vali H, Beveridge TJ, Newman DK (2004) Magnetosome vesicles are present before magnetite formation, and MamA is required for their activation. Proc. Natl. Acad. Sci. USA 101: 3839–3844. 10.1073/pnas.0400391101 15004275 PMC374331

[48] Liu T, Zhang Y, Shao H, Li X (2003) Synthesis and characteristics of Sm_2_O_3_ and Nd_2_O_3_ nanoparticles. Langmuir 19: 7569–7572.

[49] Andres Y, Maccordick HJ, Hubert J-C (1993) Adsorption of several actinide (Th, U) and lanthanide (La, Eu, Yb) ions by *Mycobacterium smegmatis*. Appl. Microbiol. Biotechnol. 39: 413–417.

[50] Faivre D, Schüler D (2008) Magnetotactic bacteria and magnetosomes. Chem. Rev. 108: 4875–4898. 10.1021/cr078258w 18855486

[51] Komeili A, Li Z, Newman DK, Jensen DJ (2006) Magnetosomes are cell membrane invaginations organized by the actin-like protein MamK. Science 311: 242–245. 10.1126/science.1123231 16373532

[52] Song H, Li X, Sun J, Yin X, Wang Y, Wu Z (2007) Biosorption equilibrium and kinetics of Au (III) and Cu (II) on magnetotactic bacteria. Chin. J. Chem. Eng. 15: 847–854.

[53] Song H, Li X, Sun J, Xu S, Hua X (2008) Application of a magnetotactic bacterium, *Stenotrophomonas* sp. to the removal of Au (III) from contaminated wastewater with a magnetic separator. Chemosphere 72: 616–621. 10.1016/j.chemosphere.2008.02.064 18439649

[54] Wang Y, Gao H, Sun J, Li J, Su Y, Ji Y, et al (2011) Selective reinforced competitive biosorption of Ag (I) and Cu (II) on *Magnetospirillum gryphiswaldense*. Desalination 270: 258–263.

[55] Xie J, Chen K, Chen X (2009) Production, modification and bioapplications of magnetic nanoparticles gestated by magnetotactic bacteria. Nano Res. 23: 261–278. 10.1007/s12274-009-9025-8 PMC2902887 20631916

[56] Tanaka M, Arakaki A, Staniland S, Matsunaga T (2010) Simultaneously discrete biomineralization of magnetite and tellurium nanocrystals in Magnetotactic bacteria. Appl. Environ. Microbiol. 76: 5526–5532. 10.1128/AEM.00589-10 20581185 PMC2918970

[57] Xu H, Xu Y, Pang X, He Y, Jung J, Xia H, et al (2015) A general route to nanocrystal kebabs periodically assembled on stretched flexible polymer shish. Science Advances 1: e1500025 10.1126/sciadv.1500025 26601151 PMC4643824

[58] Yang D, Pang X, He Y, Wang Y, Chen G, Wang W, et al (2015) Precisely size-tunable magnetic/plasmonic core/shell nanoparticles with controlled optical properties. Angew. Chem. Int. Ed. 54:12091–12096. 10.1002/anie.201504676 26331483

[59] Pang X, He Y, Jung J, Lin Z (2016) 1D nanocrystals with precisely controlled dimensions, compositions, and architectures. Science 353:1268–1272. 10.1126/science.aad8279 27634531

[60] Jabeen F, Najam-ul-Haq M, Rainer M, Güzel Y, Huck CW, Bonn GK (2015) Newly fabricated magnetic lanthanide oxides core-shell nanoparticles in phosphoproteomics. Anal. Chem. 87: 4726–4732. 10.1021/ac504818s 25859614

