# Supplementary figures and images for: Formation of Core-Shell Nanoparticles Composed of Magnetite and Samarium Oxide in Magnetospirillum magneticum Strain RSS-1
Source: PLoS One. 2017 Jan 26;12(1):e0170932. doi: 10.1371/journal.pone.0170932 (PMC5268705; doi:10.1371/journal.pone.0170932)

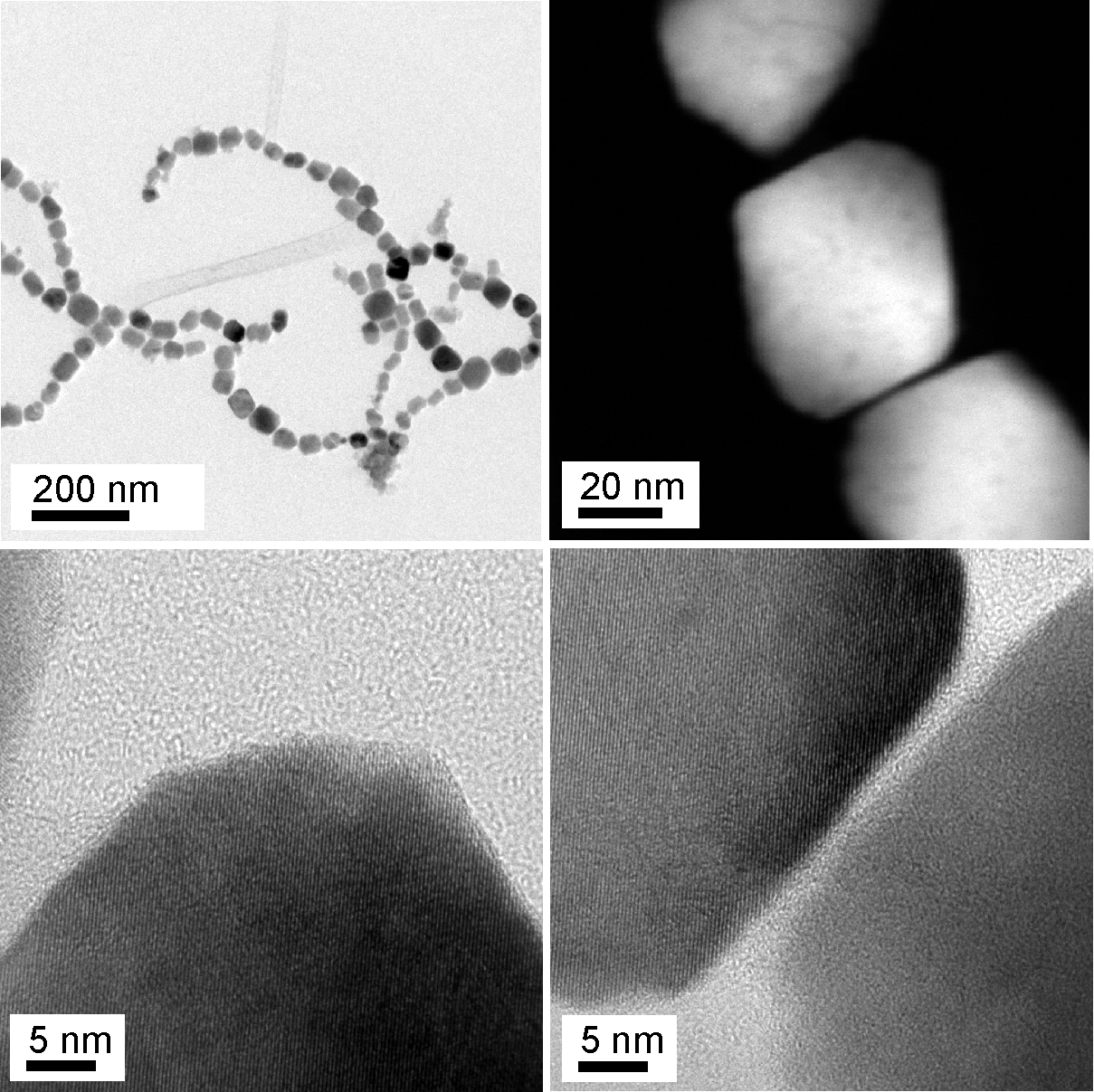

Supplement: S1 Fig — Magnetic nanoparticles were extracted from strain RSS-1 grown in the presence of only 250 μM Fe-q. (TIF) [file pone.0170932.s001.tif]

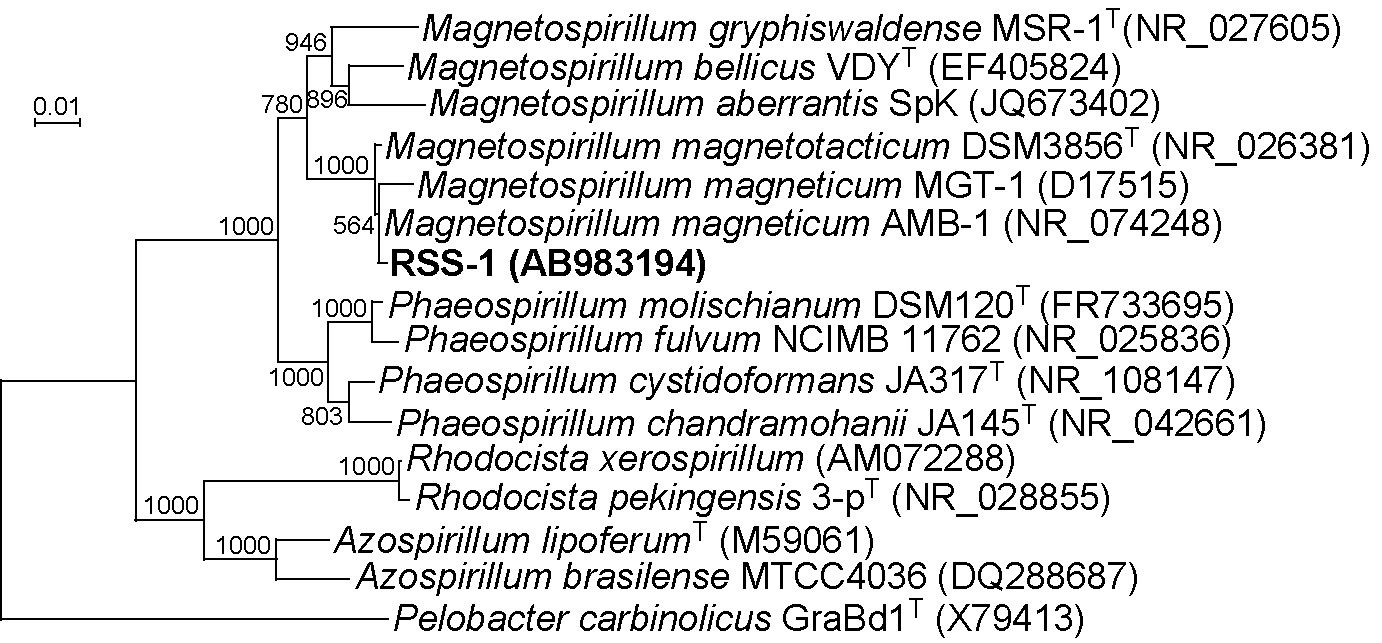

Supplement: S2 Fig — Neighbor-joining phylogenetic tree constructed based on the 16S rRNA gene sequences. The position of strain RSS-1 and some other related Magnetospirillum are represented. Bootstrap values per 1,000 replicates are indicated. The GeneBank accession numbers are shown in parentheses. Bar, 0.01 changes per nucleotide position. (TIF) [file pone.0170932.s002.tif]

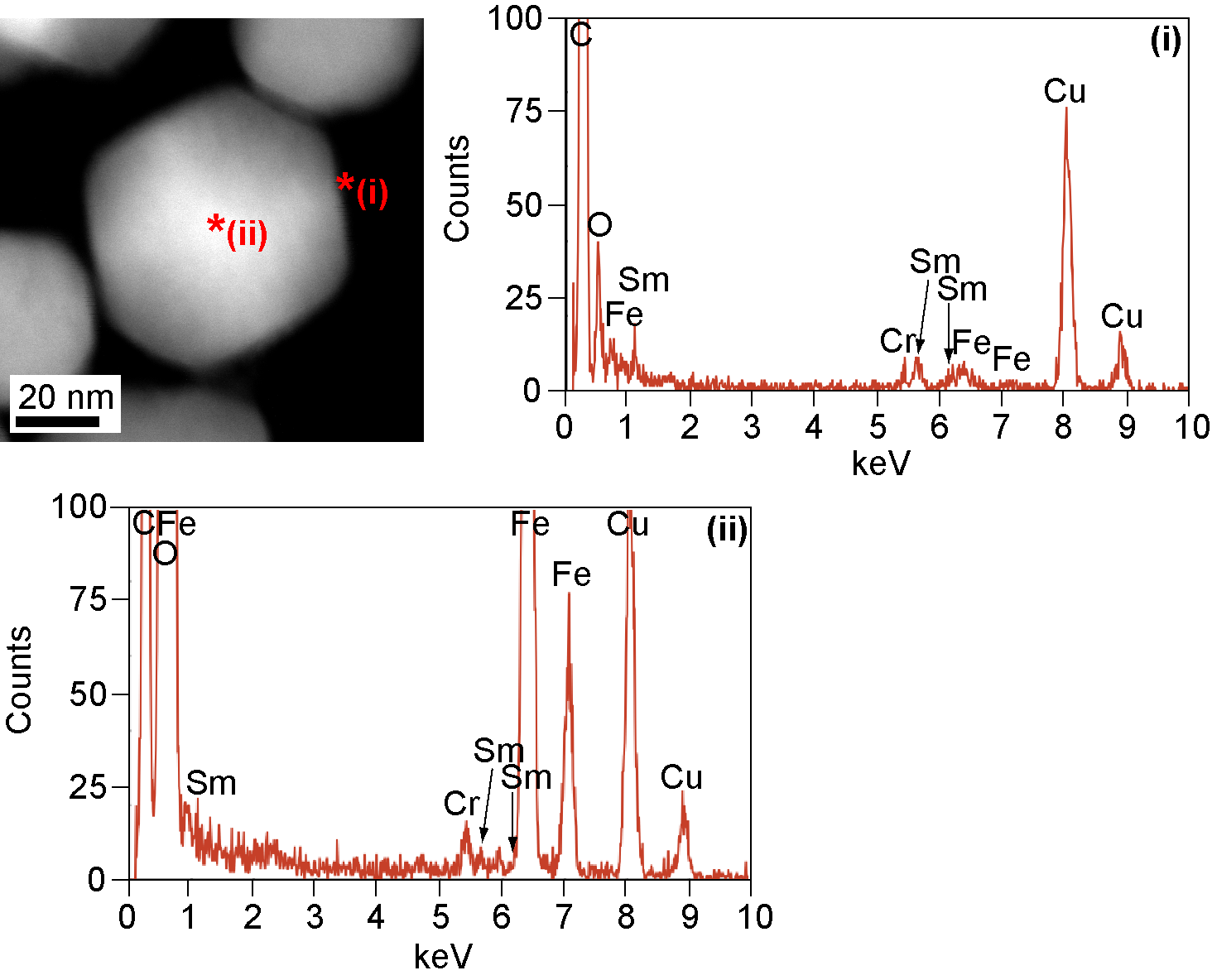

Supplement: S3 Fig — STEM-EDS spot analysis of peripheral (i) and central (ii) areas of a magnetic nanoparticle indicated by asterisks is shown. (TIF) [file pone.0170932.s003.tif]

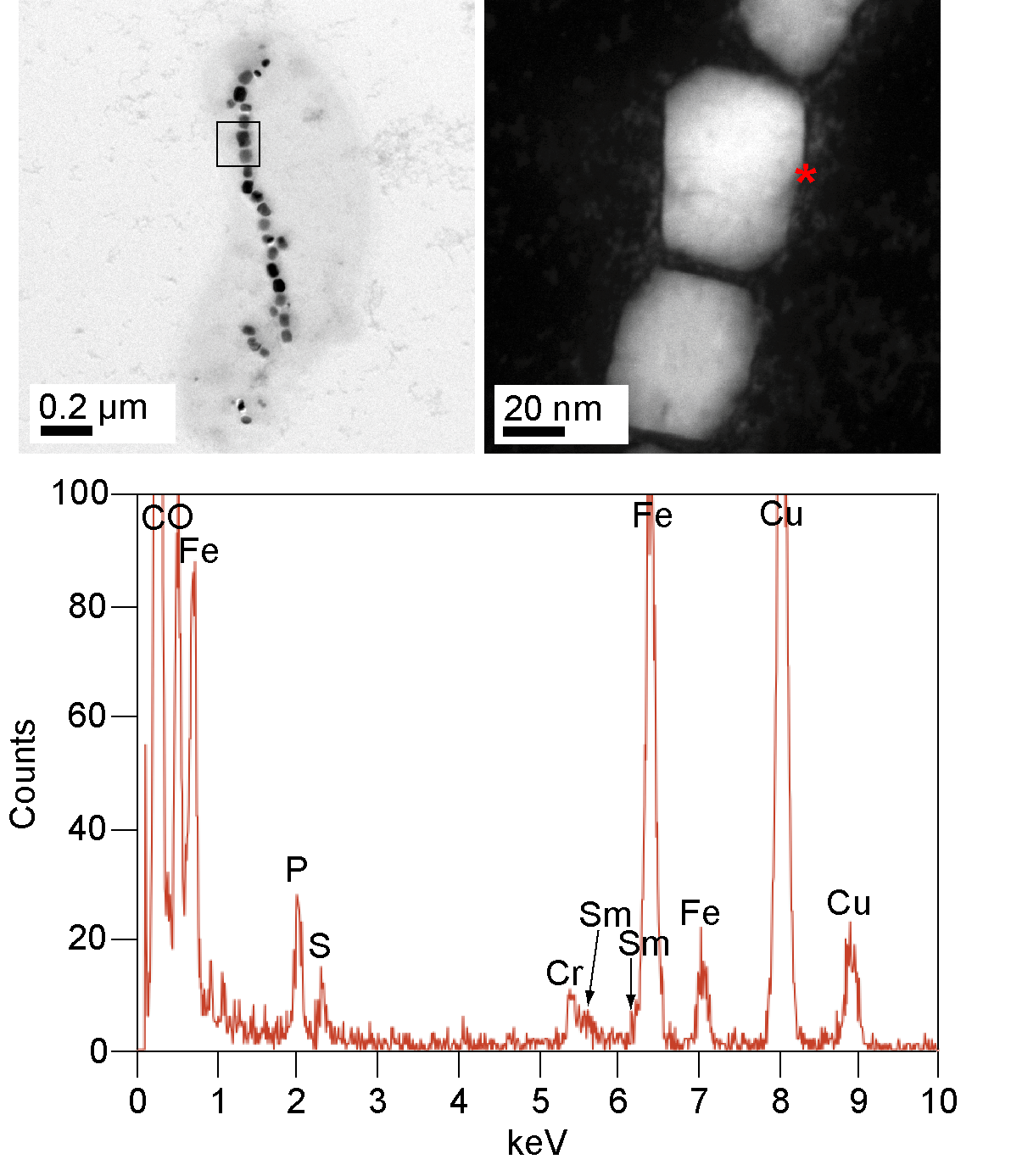

Supplement: S4 Fig — STEM-EDS spot analysis of a peripheral area of a magnetic nanoparticle indicated by an asterisk is shown. Cu signals are due to the TEM grid used, whereas the Cr signal is attributed to Cr plating on the sample holder. (TIF) [file pone.0170932.s004.tif]

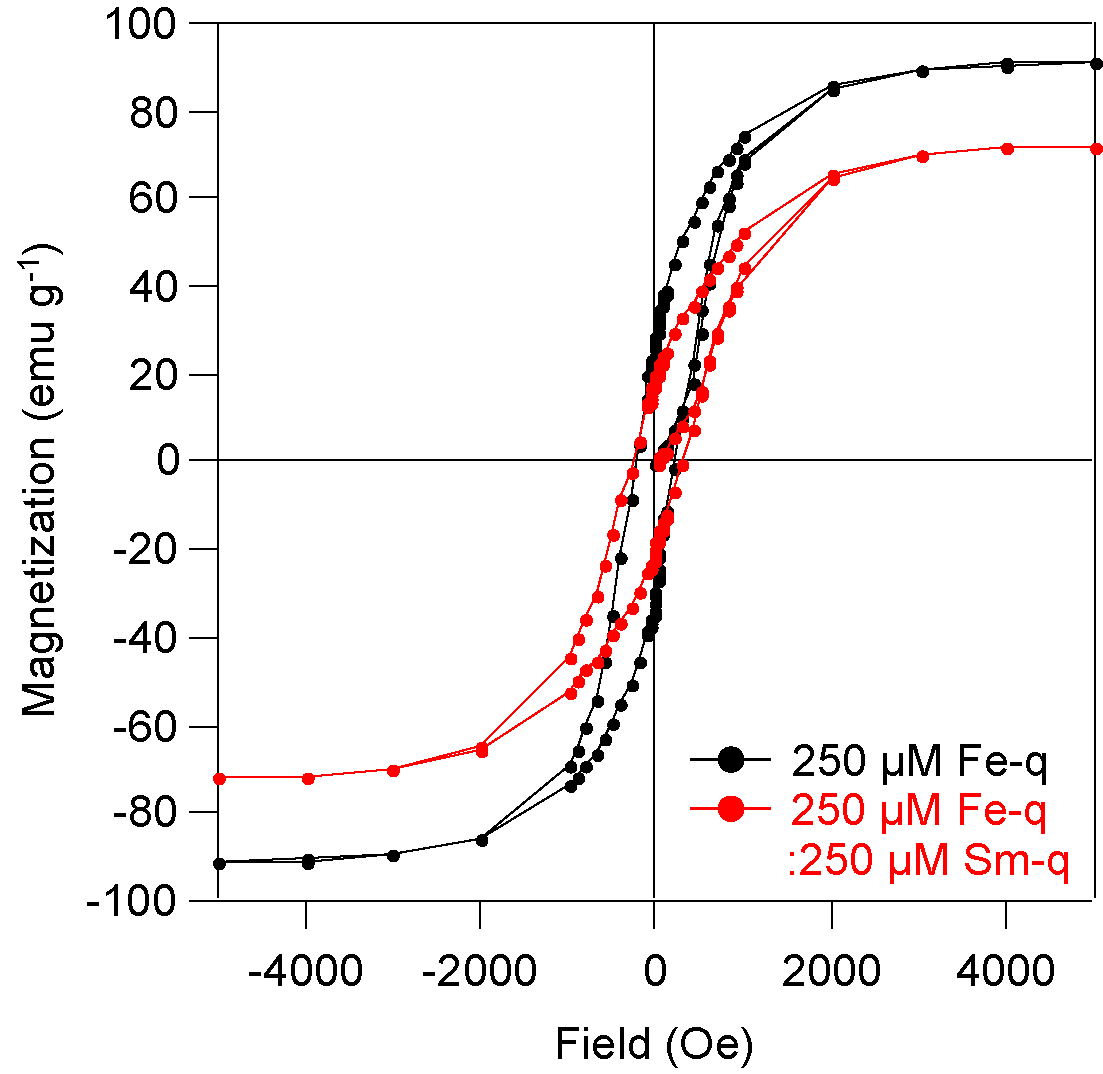

Supplement: S5 Fig — The black and red circles represent Fe3O4 NPs and Fe3O4@Sm2O3 core-shell NPs. (TIF) [file pone.0170932.s005.tif]

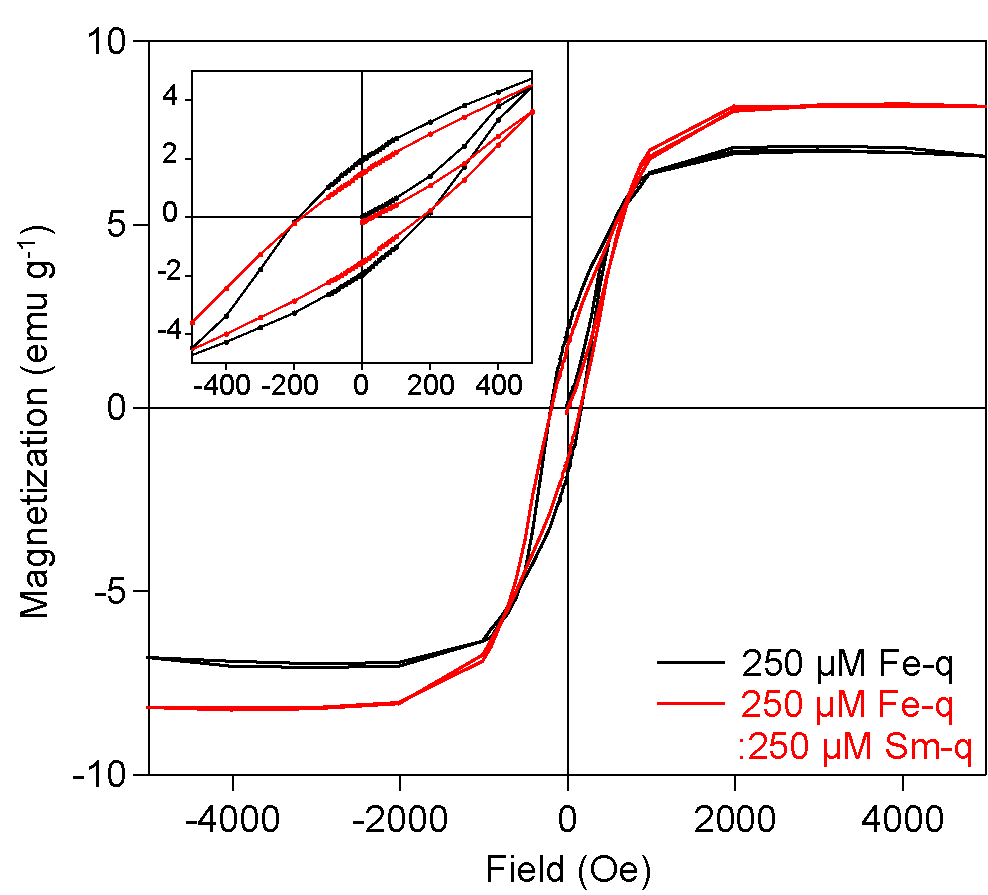

Supplement: S6 Fig — The black and red lines represent the mass magnetization in the absence and in the presence of samarium. The mass magnetization was measured at 300 K. (TIF) [file pone.0170932.s006.tif]

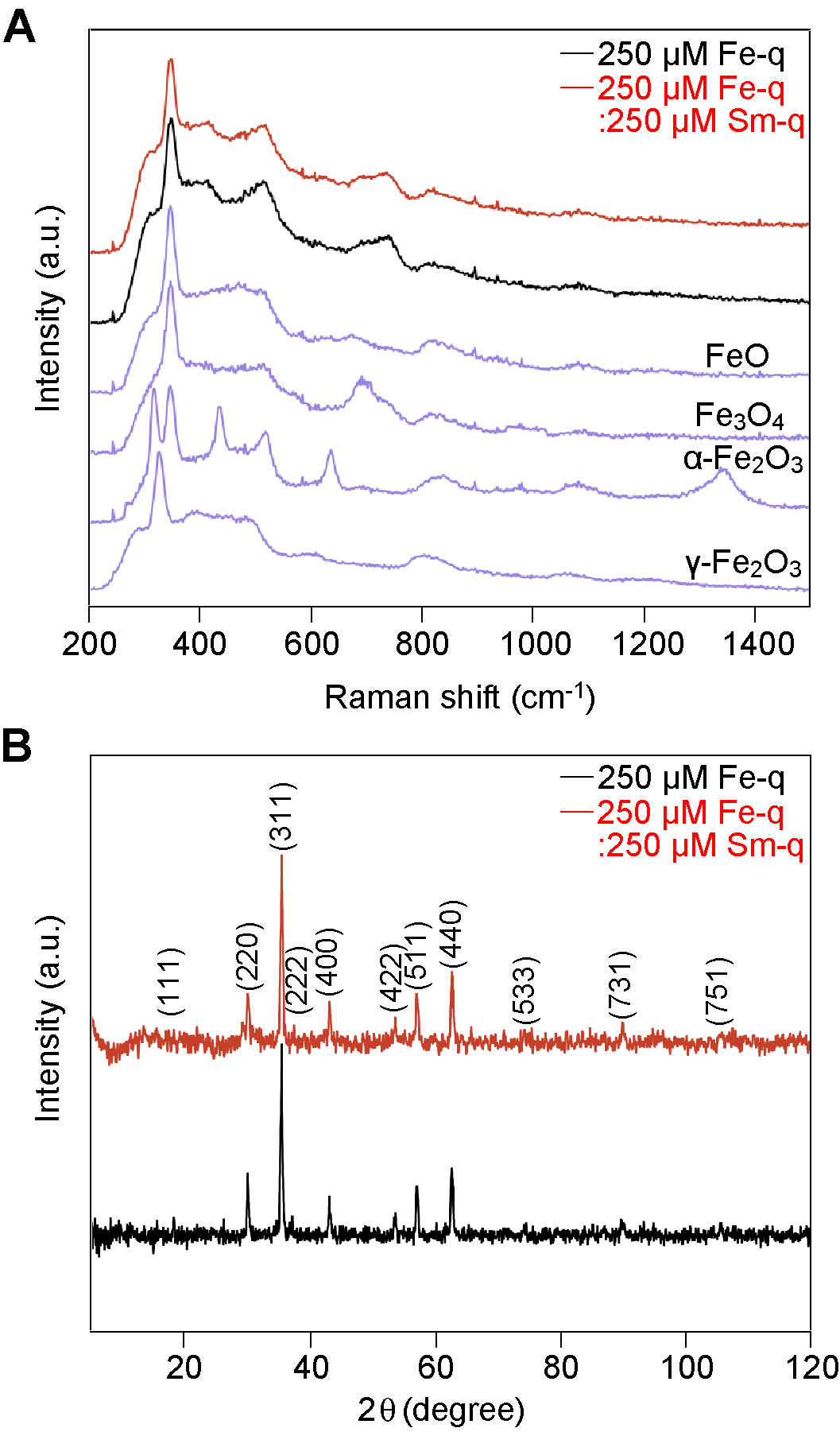

Supplement: S7 Fig — (a) Raman spectrum of the magnetite crystals with an excitation wavelength of 325 nm. The spectra corresponding to FeO, Fe3O4, α-Fe2O3 and γ-Fe2O3 are represented as references. (b) XRD of Fe3O4@Sm2O3 core-shell NPs and Fe3O4 NPs. (TIF) [file pone.0170932.s007.tif]
